# A potential mechanism for extracellular matrix induction of breast cancer cell normality

**DOI:** 10.1186/bcr3617

**Published:** 2014-02-17

**Authors:** Robert D Bruno, Gilbert H Smith

**Affiliations:** 1 School of Medical Diagnostic and Translational Sciences, College of Health Sciences, Old Dominion University, Norfolk, VA 23529, USA; 2 Mammary Stem Cell Biology Section, CCBB, Center for Cancer Research, NCI, Bethesda, MD 20892, USA

## Abstract

Extracellular matrix proteins from embryonic mesenchyme have a normalizing effect on cancer cells *in vitro* and slow tumor growth *in vivo*. This concept is suggestive of a new method for controlling the growth and spread of existing cancer cells *in situ* and indicates the possibility that extracellular proteins and/or embryonic mesenchymal fibroblasts may represent a fertile subject for study of new anti-cancer treatments.

## 

Bischof and colleagues have made a case for breast cancer cell normalization by biglycan isolated from embryonic mesenchyme *in vitro*[1]. Further, they show that injection of extracellular matrix (ECM) isolated from embryonic mesenchymal fibroblasts grown *in vitro* can suppress tumor growth when inoculated directly into growing tumors *in vivo*. Their results shed light on the potential mechanism by which embryonic mesenchyme can control tumorigenic expression of cancer cells, which was demonstrated by several investigators decades ago [2,3]. The evidence indicates that only ECM from inductive mesenchyme is effective in normalization of cancer cells. In Bischof and colleagues’ report, cancer-associated fibroblasts failed to produce a normalizing effect. The authors went on to show that tenascin C and biglycan were two ECM proteins that were uniquely expressed by inductive embryonic mesenchymal fibroblasts. Further, they demonstrated that only biglycan was able to normalize cancer cells *in vitro*. Isolated growth factors associated tightly with the purified ECM were ineffective in normalizing breast cancer cells. Finally, siRNA knockdown of biglycan mRNA and protein in embryonic fibroblasts abrogated their ability to normalize breast cancer cells or reduce their growth *in vitro*. Biglycan has been disrupted by targeting in mice, and variously leads to an osteoporosis-like phenotype and biomechanically affected specific tendons [4-6]. One wonders how the embryonic mesenchyme from these models might behave in normalization studies.

While the implication of a single ECM component, biglycan, is intriguing from a therapeutic perspective, this reductionist view of the role of ECM in controlling tumorigenesis is not in agreement with the published literature. Schedin and colleagues have very carefully studied the role of mammary ECM in breast tumorigenesis [7-10]. They have found that reproductive history and the developmental state of the gland from which the breast ECM was isolated alters the effects of the ECM on tumor growth. For instance, ECM derived from involuting mammary tissues has pro-tumor effects compared with that isolated from nulliparous tissues, and this pro-tumorigenic potential is thwarted by treatment with nonsteroidal anti-inflammatory drugs [8,9]. In addition, they report that ECM isolated from parous mammary glands has an inhibitory effect on tumor growth [7]. Contrary to Bischof and colleagues’ findings that biglycan is uniquely expressed in inductive mesenchyme, biglycan was found to be present in ECM preparations from nulliparous, parous, and involuting (with or without prior nonsteroidal anti-inflammatory drug treatment) rat mammary glands. Further, no significant differences in biglycan concentrations were identified between these ECM preparations [7,10].

Our laboratory has also been interested in trying to understand tissue-microenvironment-induced suppression of cancer cell tumorigenesis. Our data demonstrate that pluripotent human male cancer cells, mouse embryonic stem cells and breast cancer cells from both mouse and human can be induced to differentiate and not produce tumors in the mouse mammary gland during regenerative growth [11-14]. All of our published data have been determined in postpubertal mammary fat pads whose mesenchyme, according to Sakakura and colleagues [15], had lost inductive activity after interaction with mammary epithelium *in utero* at day 17. Thus, our observations and those of Schedin and colleagues do not agree with the conclusion of Bischof and colleagues that biglycan is sufficient for tumor cell normalization and that tumor cell normalization is unique to inductive mesenchyme.

The specific components involved in tumor-cell normalization are likely to be context dependent. Furthermore, changes in ECM architecture rather than ECM composition are implicated in tumor suppression by ECM isolated from mammary glands of parous rats [7], adding an additional layer of complexity to the story. Therefore, reductionist approaches to understanding these issues should be cautioned. What is clear from the whole of the published literature, including the recent report by Bischof and colleagues, is the indispensable role of the local microenvironment, including the ECM, in controlling cell growth, survival, and fate determination.

## Abbreviations

ECM: Extracellular matrix; siRNA: Small interfering RNA.

## Competing interests

The authors declare that they have no competing interests.
